# Chitinase 3-like-1 is a therapeutic target that mediates the effects of aging in COVID-19

**DOI:** 10.1172/jci.insight.148749

**Published:** 2021-11-08

**Authors:** Suchitra Kamle, Bing Ma, Chuan Hua He, Bedia Akosman, Yang Zhou, Chang-Min Lee, Wafik S. El-Deiry, Kelsey Huntington, Olin Liang, Jason T. Machan, Min-Jong Kang, Hyeon Jun Shin, Emiko Mizoguchi, Chun Geun Lee, Jack A. Elias

**Affiliations:** 1 Molecular Microbiology and Immunology,; 2 Pathology and Laboratory Medicine,; 3 Hematology-Oncology Division, Department of Medicine,; 4 The Joint Program in Cancer Biology,; 5 Cancer Center at Brown University, and; 6 Department of Biostatistics, Lifespan Health System, Warren Alpert Medical School, Brown University, Providence, Rhode Island, USA.; 7 Section of Pulmonary, Critical Care and Sleep Medicine, Department of Internal Medicine, Yale University School of Medicine, New Haven, Connecticut, USA.; 8 Department of Immunology, Kurume University, School of Medicine, Kurume, Fukuoka, Japan.; 9 Department of Medicine, Brown University, Providence, Rhode Island, USA.

**Keywords:** COVID-19, Therapeutics, Immunotherapy, Molecular pathology

## Abstract

COVID-19 is caused by SARS-CoV-2 (SC2) and is more prevalent and severe in elderly and patients with comorbid diseases (CM). Because chitinase 3-like-1 (CHI3L1) is induced during aging and CM, the relationships between CHI3L1 and SC2 were investigated. Here, we demonstrate that CHI3L1 is a potent stimulator of the SC2 receptor angiotensin converting enzyme 2 (ACE2) and viral spike protein priming proteases (SPP), that ACE2 and SPP are induced during aging, and that anti-CHI3L1, kasugamycin, and inhibitors of phosphorylation abrogate these ACE2- and SPP-inductive events. Human studies also demonstrate that the levels of circulating CHI3L1 are increased in the elderly and patients with CM, where they correlate with COVID-19 severity. These studies demonstrate that CHI3L1 is a potent stimulator of ACE2 and SPP, that this induction is a major mechanism contributing to the effects of aging during SC2 infection, and that CHI3L1 co-opts the CHI3L1 axis to augment SC2 infection. CHI3L1 plays a critical role in the pathogenesis of and is an attractive therapeutic target in COVID-19.

## Introduction

SARS-CoV-2 (SC2) is a coronavirus that was initially discovered in humans in 2019. It is highly transmissible and has caused a global pandemic, killing more than 3.8 million people and infecting many millions more worldwide since the pandemic was declared on March 11, 2020 (1–8). The disease caused by SC2, COVID-19, was initially noted to manifest as a pneumonia with fever, cough, fatigue, and dyspnea as major manifestations (9). However, it is now known to be a systemic disease with manifestations in many organs (10, 11). In addition, it is now known that there is a spectrum of disease severity, including patients who are asymptomatic, 10%–20% of patients who require hospitalization (4, 5, 12), and patients with respiratory failure who require intensive care (13). Unique clinical features have also been described, including pulmonary fibrosis, an increased frequency of vascular thrombotic events, a coagulopathy, and pulmonary angiitis (14, 15). However, the cellular and molecular events that account for this impressive clinical and pathologic heterogeneity are poorly understood.

One of the most unique features of COVID-19 is its impressive relationship to aging and comorbid disorders. The elderly are at increased risk of contracting COVID-19 and experience higher complication and case fatality rates (16, 17). COVID-19 has also had devastating effects on the elderly and residents of congregate care facilities (18, 19). Similarly, approximately 50% of hospitalized COVID-19 patients have preexisting medical conditions including diabetes, hypertension, obesity and metabolic syndrome, cardiovascular disease, and chronic lung diseases like COPD and asthma. These comorbidities associate with enhanced susceptibility to SC2 infection, more severe disease, and a higher mortality (1–3, 5, 16, 20, 21). Surprisingly, the cellular and molecular events that underlie the effects of aging and comorbid diseases in COVID-19 have not been defined.

SC2 interacts with cells via its spike (S) protein, which binds to its host cell receptor angiotensin converting enzyme 2 (ACE2); ACE2 mediates viral entry (22–25). After binding, the S protein is processed by the S priming proteases (SPP) transmembrane serine protease 2 (TMPRSS2), Cathepsin L (CTSL), and in some cases, FURIN into S1 and S2 subunits. The latter mediates the fusion of the viral envelope and cell plasma membrane, which is required for virus-cell entry (16, 20, 24). In the human disease, SC2 passes through the mucus membranes of the upper and lower respiratory tracts and infects epithelial cells via this ACE2-S protein-protease mechanism (16, 20, 26). The virus can then enter the bloodstream and, via this viremia, infect other cells and organs that express ACE2 and SPP (27). It has been proposed that the levels of expression of ACE2 and the SPP play key roles in determining the magnitude and organ location of the infection and the severity of the disease (16, 20, 28). ACE2 is expressed widely in human tissues (16), and in the lung, ACE2 is expressed at high levels in the nasopharynx and at lesser levels in the lower airways and alveolar areas (29). However, the mechanisms that SC2 uses to generate its effects in different tissues have not been defined. In addition, the regulation of ACE2 expression in pulmonary and nonpulmonary tissues and its contribution to the specific pathologies of SC2 infection have not been sufficiently investigated.

Virus infection of mammalian cells leads to innate and adaptive immune responses that restrict viral replication, augment viral clearance, and limit tissue damage and disease severity (30). To allow infection to occur, many viruses have developed strategies to evade and/or suppress these immune responses (30–32). They can also co-opt host responses to augment viral replication (30–32). These events can drastically influence disease pathogenesis, the course of the infection, disease severity, and viral persistence in the host (30–32). Compared with other positive stranded (+) RNA viruses, coronaviruses have exceptionally large genomes and employ complex genome expression strategies (30). Many of the coronavirus genes participate in virus-host interplay to create an optimal environment for viral replication (30). However, the host pathways that SC2 co-opts to foster infection and replication have not been adequately defined, and the possibility that SC2 utilization of host responses contributes to the mechanisms by which aging and comorbid diseases augment the prevalence and severity of COVID-19 has not been addressed.

SC2, like other RNA viruses, is prone to genetic evolution with the development of mutations over time (8). This results in the emergence of viral variants that have different characteristics and can manifest enhanced infectivity and immune resistance compared with ancestral strains (8). The D614G has replaced the “D” (aspartic acid) with a “G” (glycine) at position 614 of the S protein. It was initially noted during the early phases of the pandemic and subsequently evolved into a globally dominant variant (8, 33). The D614G is now known to be a common mutation that is found in all of the major SC2 variants, including the α (B.1.1.7 lineage), β (B.1.351 lineage), γ (P.1 lineage), δ (B.1.617.2 lineage), and λ (C.37 lineage) moieties (34). SC2 containing the D614G mutation is known to transmit efficiently (35). However, relationships between D614G, CHI3L1, ACE2, and SPP have not been defined.

Chitinase 3-like-1 (CHI3L1; also called YKL-40 in human and Chil1 or BRP-39 in mouse) is an evolutionarily conserved member of the 18 glycosyl hydrolase (GH 18) gene family that is produced by a spectrum of cells in response to a variety of injury and cytokine stimuli (36–40). It is the cornerstone of a critical pathway that is activated during injury and inflammation, regulates innate and adaptive immunity, and heals and protects (37, 39, 41–43). The latter is mediated by its ability to inhibit apoptosis and other forms of cell death while driving fibroproliferative repair (41, 43). CHI3L1 is readily detected in the circulation of healthy individuals, and the levels of circulating and tissue CHI3L1 are increased in diseases characterized by inflammation, injury, remodeling, and repair (36–38, 42, 44–51). Interestingly, CHI3L1 is also expressed in an exaggerated manner in aging and in the same comorbid diseases that are risk factors for COVID-19 (52–59). CHI3L1 and ACE2 are also both mediators of pulmonary protective responses (41, 60–63). In light of these impressive similarities, we hypothesized that the induction of ACE2 and SPP is part of the CHI3L1-induced healing and repair response. We also hypothesized that SC2 co-opts the host CHI3L1 axis to augment its ability to infect, spread and generate disease. To address these hypotheses, we used genetically modified mice, in vitro approaches, and human cohorts. These studies demonstrated that (a) CHI3L1 is a potent stimulator of ACE2 and SPP in pulmonary epithelial cells and vascular cells; (b) the expression of ACE2 and SPP are increased during murine aging and that these aging-induced inductive events are mediated by CHI3L1; and (c) interventions that alter CHI3L1 effector responses are also potent inhibitors of ACE2 and SPP and viral infection. Lastly, human studies demonstrated that the levels of circulating CHI3L1 are increased in COVID-19^+^ patients who are elderly, have comorbid diseases, and manifest severe COVID-19 disease. These findings support the concept that CHI3L1 plays a critical role in the pathogenesis of and is an attractive therapeutic target in COVID-19. They also provide a mechanistic explanation for how aging and comorbid diseases contribute to the pathogenesis of COVID-19.

## Results

### CHI3L1 regulation of pulmonary ACE2 and SPP in vivo.

**Figure 1:**
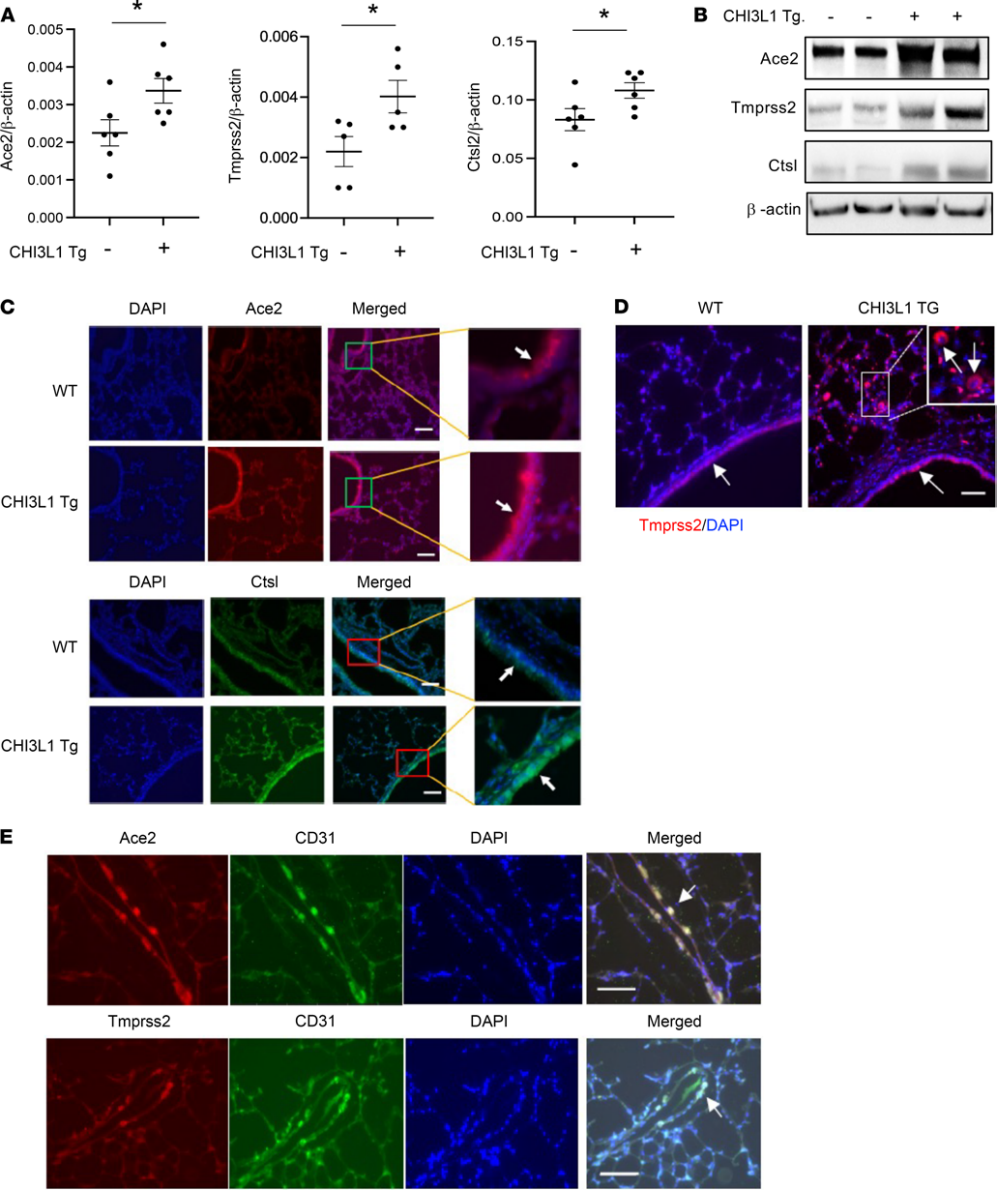
CHI3L1 stimulates pulmonary ACE2 and SPP. Eight-week-old WT (–) and *CHI3L1* Tg (+) mice were sacrificed after 2 weeks of transgene induction with doxycycline. Levels of Ace2 and SPP mRNA and protein in lungs from WT and *CHI3L1* Tg mice were evaluated using lung lysates and paraffin tissue blocks. (**A**) Comparisons of expression levels of ACE2 and SPP in lungs from WT (Tg^–^) and *CHI3L1* Tg^+^ mice using semiquantitative real-time reverse transcription PCR (RT-qPCR) indexed to β-actin controls. (**B**) Western immunoblot comparisons of ACE2 and SPP levels in lungs from WT (Tg^–^) and *CHI3L1* Tg^+^ mice. (**C** and **D**) IHC of ACE2, CTSL, and TMPRSS2 in lungs from WT and *CHI3L1* Tg mice. (**E**) Double-label IHC comparing localization of ACE2, TMPRSS2, and CD31 in lungs from *CHI3L1* Tg mice. Arrows on **C**–**E** indicate stain^+^ cells. Each value in **A** is from a different animal; mean ± SEM is illustrated. **B**–**E** are representative of at least 3 separate evaluations. β-Actin was used as an internal control. ACE2, murine angiotensin converting enzyme 2; TMPRSS2, transmembrane serine protease 2; CTSL, Cathepsin L. Scale bars: 100 μm. *P < 0.05 (Student *t* test).

To begin to address the relationship between CHI3L1 and SC2, we characterized the effects of CHI3L1 on the expression and accumulation of ACE2 and SPP in vivo. In these studies, we compared the expression of Ace2 and SPP in lungs from inducible CC10 promoter–driven CHI3L1-overexpressing Tg mice (CHI3L1 Tg) and WT controls. These studies revealed the induction of ACE2, TMPRSS2, and CTSL in lungs from CHI3L1 Tg mice (Figure 1, A and B). ACE2 protein was most prominent in airway epithelium, where it colocalized with CC10 (Figure 1C and Supplemental Figure 1A; supplemental material available online with this article; https://doi.org/10.1172/jci.insight.148749DS1). ACE2 was also seen in alveoli, where it colocalized with pro–SP-C (Supplemental Figure 1B). TMPRSS2 and CTSL were also prominently expressed in airway epithelial cells and, to a lesser extent, alveolar epithelial cells (Figure 1, C and D, and Supplemental Figure 1, C–F), and TMPRSS2 was also seen in alveolar macrophages (Figure 1D). CHI3L1 Tg also augmented the expression and accumulation of ACE2 and SPP in pulmonary blood vessels. IHC demonstrated that ACE2 and TMPRSS2 colocalized with CD31 on endothelial cells and TRANSGELIN^+^ vascular smooth muscle cells (Figure 1E and Supplemental Figure 1G).

To define the importance of endogenous CHI3L1, we also compared the expression of ACE2 and SPP in lungs from WT and CHI3L1-null mutant mice. These studies demonstrated that the expression of ACE2 and SPP was decreased in lungs from mice that lack CHI3L1 (Supplemental Figure 2). When viewed in combination, these studies demonstrate that endogenous and exogenous CHI3L1 are potent stimulators of epithelial and vascular cell ACE2 and SPP in vivo.

### CHI3L1 regulation of pulmonary ACE2 and SPP in vitro.

**Figure 2:**
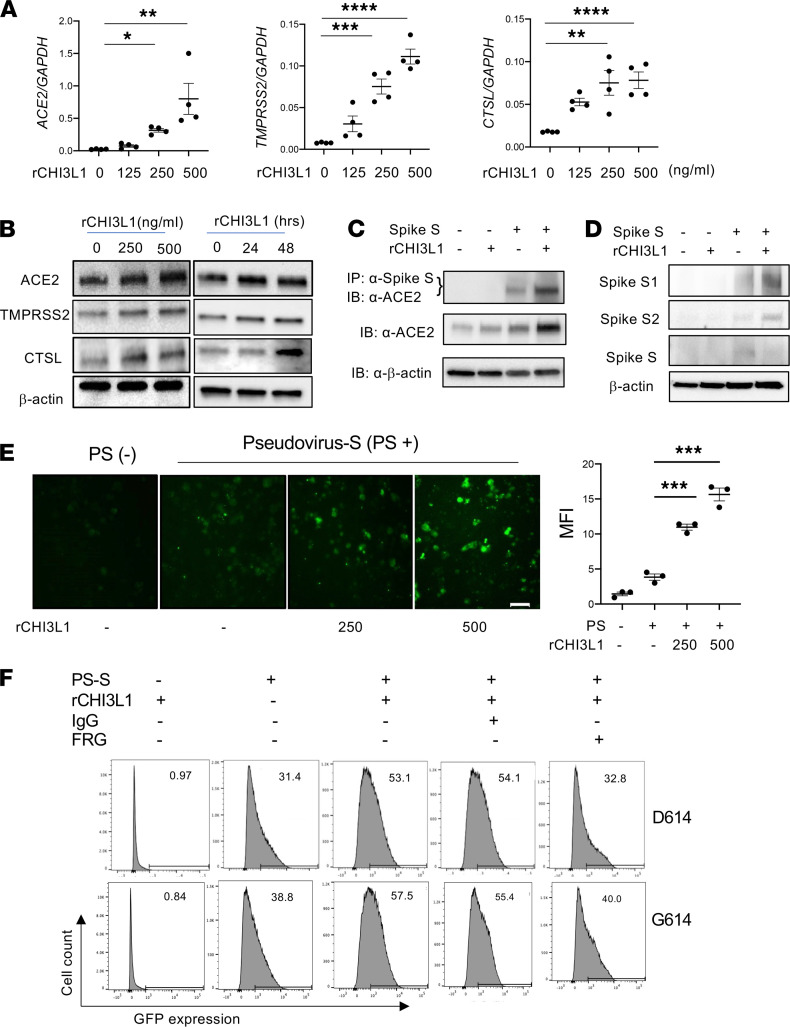
CHI3L1 stimulates ACE2 and SPP in vitro and enhances S protein processing and cellular integration. (**A**) Calu-3 lung epithelial cells were incubated with the noted concentrations of recombinant CHI3L1 (rCHI3L1; ng/mL) or vehicle control (rCHI3L1 = 0) for 24 hours and were then subjected to RT-qPCR to quantitate the levels of mRNA encoding ACE2 and the SPP. (**B**) Western blot evaluations of the dose response and kinetics of CHI3L1 stimulation of ACE2 and SPP protein accumulation in Calu-3 cells. (**C** and **D**) Calu-3 cells were incubated with vehicle (PBS; CHI3L1^–^) or rCHI3L1 (500 ng/mL) for 24 hours, recombinant S protein of SC2 was added, and the incubation continued for an additional 2 hours. The cell lysates were prepared, Co-IP and immunoblot assays were undertaken (**C**), and the uncleaved S and cleaved S1 and S2 proteins were evaluated using Western immunoblotting (**D**). (**E**) Calu-3 cells were incubated with vehicle (rCHI3L1^–^) or the noted concentrations of rCHI3L1 for 24 hours. They were then transfected with a pseudovirus containing the S protein (PS; D614 variant) from SC2 and a GFP expression construct and incubated for additional 24 hours and then evaluated using fluorescence microscopy. Quantification of mean fluorescence intensity (MFI) can be seen in the dot plot on the right. (**F**) Calu-3 cells were incubated with rCHI3L1 or vehicle control for 48 hours in the presence or absence of antibody against CHI3L1 (FRG) or control antibody (IgG). Pseudovirus S (PS; D614 and G614 variants) that expressed GFP was added as noted, and GFP-positive cells (%) were evaluated by flow cytometry. Values in **A** and **E** are mean ± SEM. **B**–**F** are the representative data obtained from at least 3 independent experiments. β-Actin or GAPDH was used as an internal control. *P < 0.05, **P < 0.01, ***P < 0.001, ****P < 0.0001 (1-way ANOVA with post hoc Dunnett’s multiple comparison test). Scale bar: 50 μm.

In vitro experiments were next undertaken to determine if rCHI3L1 regulated the expression and/or accumulation of ACE2 and SPP. These studies demonstrated that rCHI3L1 stimulated ACE2 and SPP (TMPRSS2, CTSL) gene expression and protein accumulation in human Calu-3 epithelial cells in dose- and time-dependent manners (Figure 2, A and B). These effects were not Calu-3 cell specific because similar results were obtained with A549 epithelial cells, primary human small airway epithelial cells (HSAECs), and lung fibroblasts (Supplemental Figures 3 and 4). When viewed in combination, these studies demonstrate that CHI3L1 stimulates ACE2 and SPP in a variety of cells in vitro.

### Consequences of ACE2 and SPP induction.

To understand the consequences of CHI3L1 induction of ACE2 and SPP, studies were undertaken to determine if this induction augmented the ACE2 receptor binding of S protein and/or the metabolism of S into its S1 and S2 subunits. In these experiments, lysates were prepared from Calu-3 cells incubated with rCHI3L1 or vehicle control, and recombinant S (rS) protein was added. To assess S-receptor binding, IP and immunoblots were serially undertaken with anti-ACE2 and anti-S, respectively. To assess S metabolism, the cell lysates were incubated for 24 hours with rS, and Western blots were undertaken with S1- and S2-specific antibodies. As can be seen in Figure 2, C and D, CHI3L1 stimulation was associated with enhanced ACE2-S binding and enhanced metabolism of S into its respective subunits.

To further address the consequences of these inductive events, pseudovirus moieties were generated by incorporating the SC2 S protein into lentivirus moieties that contained a GFP marker. The infectious capacity of these pseudovirus S moieties was then evaluated using cells incubated with rCHI3L1 or control vehicle. In keeping with the findings noted above, rCHI3L1 induction of ACE2 and SPP augmented pseudovirus S incorporation into lung epithelial cells (Figure 2E). FACS evaluations further confirmed these observations by demonstrating that rCHI3L1 enhanced the cellular integration of S protein–containing pseudovirus and that treatment with anti-CHI3L1 monoclonal antibody (FRG) ameliorated rCHI3L1-stimulated pseudovirus cell integration (Figure 2F). CHI3L1 similarly enhanced the cellular integration of pseudovirus containing S sequence with D614 or G614, and FRG treatment inhibited pseudoviral infection regardless of S sequence (Figure 2F). When viewed in combination, these studies demonstrate that CHI3L1 stimulation of ACE2 and SPP increases S-ACE2 binding, S metabolism, and pseudovirus S infection.

### Monoclonal and small molecule CHI3L1–based therapeutics.

**Figure 3:**
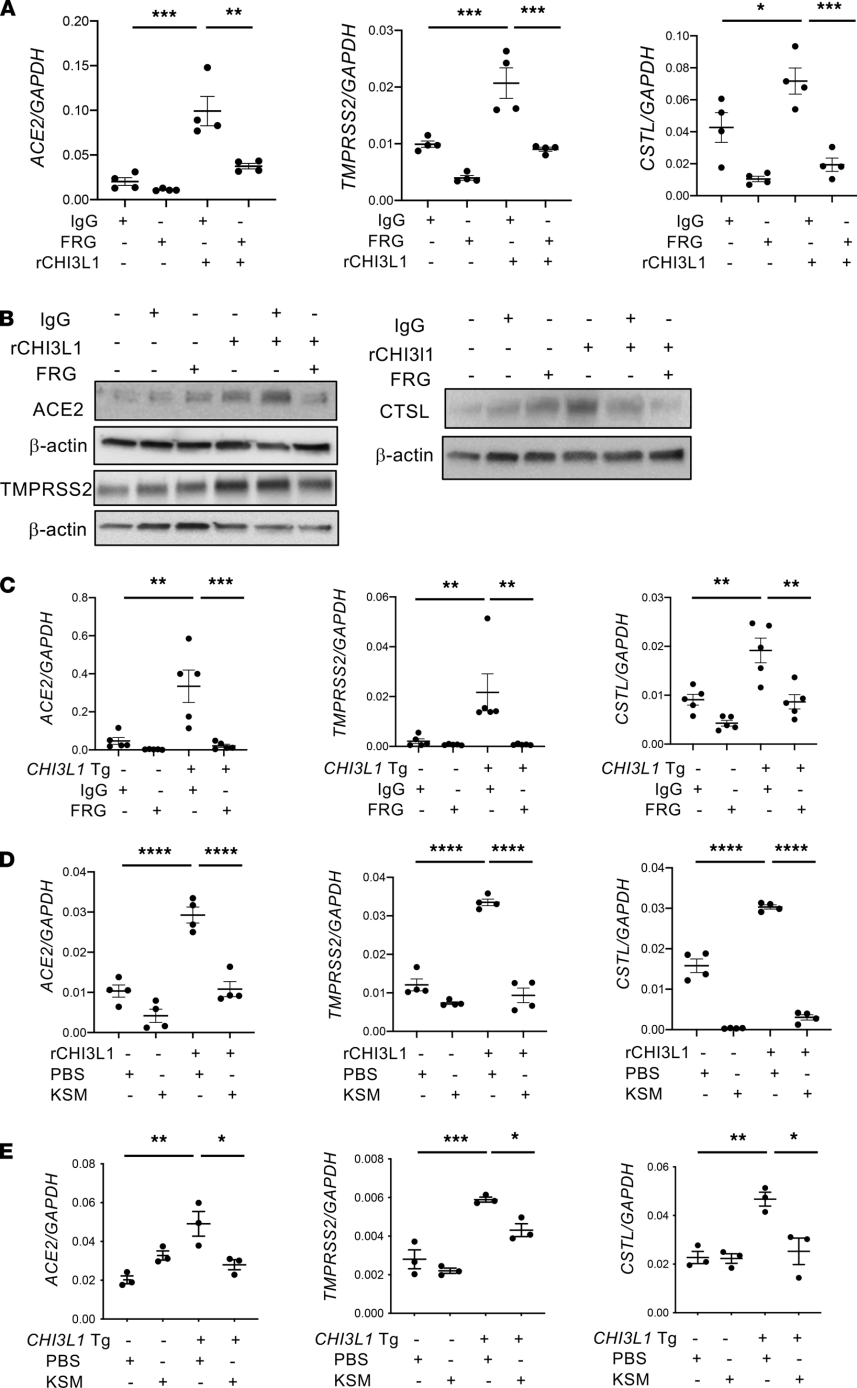
Effects of a monoclonal antibody and small molecule inhibitor on CHI3L1 stimulation of ACE2 and SPP in vitro and in vivo. (**A** and **B**) Calu-3 cells were stimulated with vehicle (PBS) or rCHI3L1 (250 ng/mL) and treated with isotype control IgG or anti-CHI3L1 (FRG) for 48 hours. The cells were then harvested, and the levels of mRNA encoding ACE2 and SPP, and ACE2 and SPP protein, were evaluated by RT-qPCR (**A**) and immunoblot assays (**B**). (**C**) WT and *CHI3L1* Tg mice were treated with IgG isotype control antibody or FRG antibody during their 2 weeks of transgene activation (i.p. every other day, 200 μg/mouse). The levels of mRNA encoding *Ace2* and SPP were then evaluated using RT-qPCR. (**D**) Calu-3 cells were stimulated with vehicle (PBS) or rCHI3L1 (250 ng/mL) and treated with kasugamycin (250 ng/mL) or vehicle control (PBS) for 48 hours. The cells were then harvested, and the levels of mRNA encoding ACE2 and SPP were evaluated by RT-qPCR. (**E**) WT and *CHI3L1* Tg mice were treated with vehicle (PBS) or kasugamycin (50 mg/kg/mouse, i.p. every other day) during their 2 weeks of transgene activation. The levels of mRNA encoding Ace2 and SPP were then evaluated using RT-qPCR. β-Actin or GAPDH was used as an internal control. Each value in **A**, **C**, and **E** is from a different animal, and the mean ± SEM is illustrated. **B** is representative of 2 independent experiments. *P < 0.05, **P < 0.01, ***P < 0.001, ****P < 0.0001 (1-way ANOVA with post hoc Tukey multiple comparison test).

The finding that CHI3L1 is a potent stimulator of ACE2 and SPP raises the interesting possibility that CHI3L1 may be a useful therapeutic target in COVID-19. To begin to address this possibility, we characterized the effects of a humanized monoclonal antibody against CHI3L1 developed in our laboratory (called FRG), as well as a small molecule Chitinase 1 and CHI3L1 inhibitor called kasugamycin in vitro and in vivo. The monoclonal inhibitor of CHI3L1 (FRG) was a potent inhibitor of CHI3L1 stimulation of epithelial cell ACE2 and SPP in vitro (Figure 3, A and B). FRG was also a powerful inhibitor of CHI3L1 Tg induction of ACE2 and SPP in vivo (Figure 3C). Interestingly, kasugamycin was a similarly powerful inhibitor of CHI3L1 induction of ACE2 and SPP in vitro and in vivo (Figure 3, D and E). These studies highlight antibody-based and small molecule CHI3L1 inhibitors that control ACE2 and SPP and have promise as therapeutics in COVID-19.

### CHI3L1 phosphorylation–based therapeutics.

**Figure 4:**
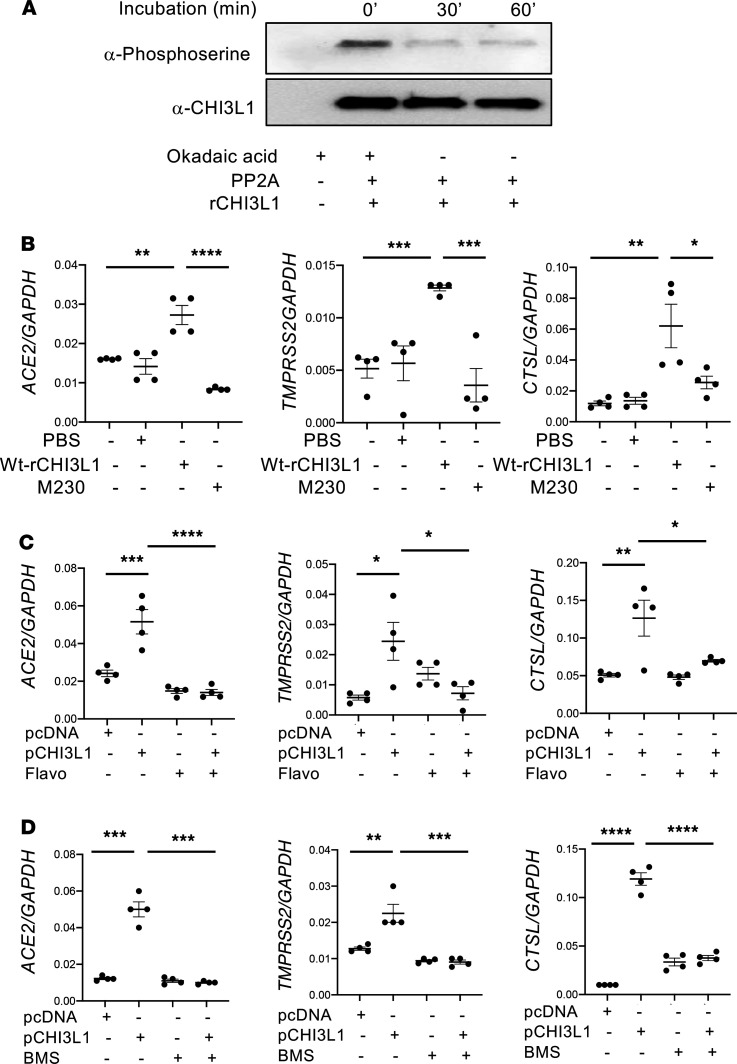
Alterations in CHI3L1 phosphorylation modify its ability to regulate ACE2 and SPP. (**A**) Demonstration that CHI3L1 is a phosphoprotein. Biologically active rCHI3L1 was treated with protein phosphatase 2A (PP2A) in the presence and absence of the PP2A inhibitor Okadaic acid. The alterations in CHI3L1 phosphorylation were evaluated with immunoblots using antibodies against phosphoserine or CHI3L1 controls. (**B**) Calu-3 cells were stimulated with a WT or a mutant form of rCHI3L1 (250 ng/mL for each; 24 hours) that could not be phosphorylated. The latter was done by generating a serine to arginine mutation at aa 230. The cells were then harvested, and the levels of mRNA encoding ACE2 and SPP2 was evaluated by RT-qPCR. (**C** and **D**) Calu-3 cells were transfected with pcDNA (vector only) or the plasmid containing a CHI3L1 cDNA (pCHI3L1). They were simultaneously treated with flavopiridol (Flavo; 5 nM) (**C**) or BMS 265246 (BMS; 9 nM) (**D**) or their vehicle controls (5% DMSO) for 24 hours. The cells were then harvested, and the levels of mRNA encoding ACE2 and SPP were evaluated by RT-qPCR. **A** is representative immunoblots in 3 separate experiments. GAPDH was used as an internal control, The values in **B** and **D** are mean ± SEM. Each value in **C** is from a different animal, and the mean ± SEM is illustrated. *P < 0.05, **P < 0.01, ***P < 0.001, ****P < 0.0001 (1-way ANOVA with post hoc Tukey multiple comparison test).

To date, all studies of CHI3L1 have assumed that it is not posttranslationally modified. However, phosphorylation site prediction analysis using the NetPhos program (v2.0) suggests that CHI3L1 is a phosphoprotein with high potential for serine/threonine phosphorylation. Further investigation also provided a number of lines of evidence that suggest that CHI3L1 is effective as a phosphoprotein. This included (a) sequence mining of CHI3L1, which revealed a cyclin binding domain that is highly predictive of cyclin-dependent kinase (CDK) phosphorylation (Supplemental Figure 5); (b) sites of serine phosphorylation of rCHI3L1 confirmed with immunoblot assays with an antiphosphoserine antibody (EMD Millipore, AB1603) (Figure 4A); and (c) the demonstration that CHI3L1 phosphorylation is dependent on CDK activity based on its inhibition by the broad spectrum CDK inhibitor flavopiridol. As a result, we hypothesized that phosphorylation in this region is essential for CHI3L1 effector responses. To address this hypothesis, we used in vitro and in vivo approaches. In the former, we generated WT CHI3L1 and mutant forms of rCHI3L1 that could not be phosphorylated and compared their ability to induce epithelial cell ACE2 and SPP. As can be seen in Figure 4B, WT CHI3L1 was a powerful stimulator of epithelial ACE2 and SPP, and this effect was abrogated by mutations at aa 230 (serine to arginine mutation at site of aa 230 of CHI3L1). Mutations at aa 235 or 237 did not have similar effects (data not shown). In the in vivo experiments, we compared the effects of CHI3L1 Tg in mice treated with the broad spectrum CDK inhibitor flavopiridol (Figure 4C) or the selective CDK 1 and 2 inhibitor (BMS 265246) and their vehicle controls (Figure 4D). In both cases, the CDK inhibitors abrogated the ability of CHI3L1 to stimulate epithelial cells ACE2 and SPP. These studies demonstrate that CHI3L1 is effective as a phosphoprotein, and they highlight the ability of CDK inhibitors, particularly inhibitors of CDK 1 and 2, to control this phosphorylation and, in turn, serve as a therapeutic in COVID-19.

### CHI3L1 and aging in COVID-19.

**Figure 5:**
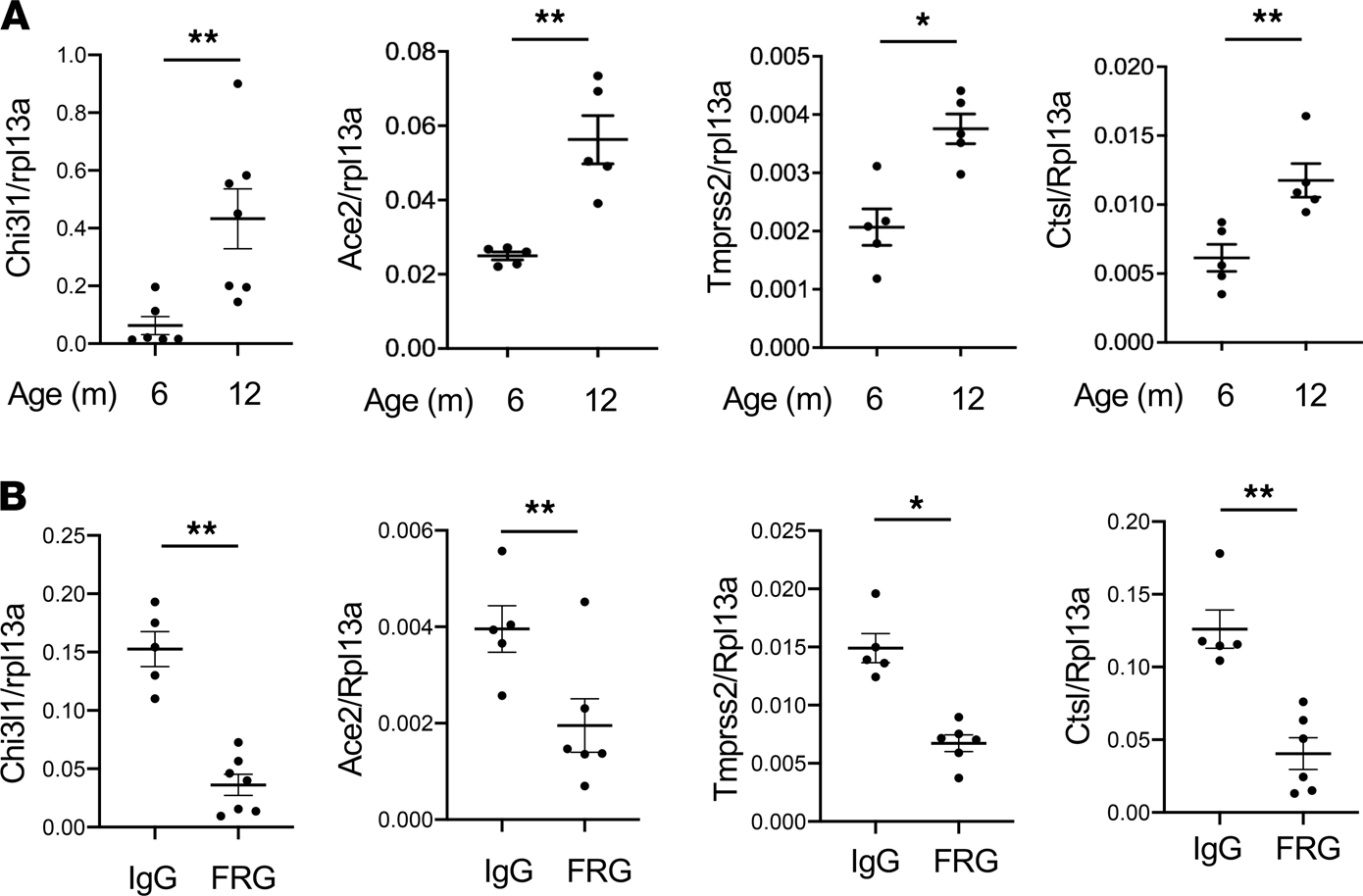
CHI3L1 is induced and regulates the expression of ACE2 and SPP in the lungs of aged mice. (**A**) Comparison of the levels of mRNA encoding CHI3L1, ACE2, and SPP in lungs from 6- and 12-month-old WT mice evaluated by RT-qPCR. (**B**) WT mice were treated with IgG isotype antibody or anti-CHI3L1 (FRG) (200 μg/mouse, twice a week, i.p. injection) when the mice were between 6 and 12 months of age. At the end of this interval, the mice were sacrificed, and the levels of mRNA encoding pulmonary CHI3L1, ACE2, and SPP were evaluated by RT-qPCR. Ribosomal protein L13a (Rpl13a) was used as an internal control. Each value in **A** and **B** is from a different animal, and the mean ± SEM is illustrated. *P < 0.05, **P < 0.01 (Mann-Whitney *U* test).

The elderly are at increased risk of contracting COVID-19 and experience higher complication and case fatality rates (16, 17). These unique features have interesting parallels in the CHI3L1 axis, where the levels of CHI3L1 expression are known to increase with aging (64). To further understand these relationships, we measured the levels of CHI3L1 expression in WT mice as they age. As can be seen in Figure 5A, the expression of CHI3L1 and its downstream ACE2 and SPP expression were increased significantly in comparisons of 6- and 12-month-old WT mice. Importantly, treatment of WT mice twice a week with the monoclonal antibody FRG from 6 months to 12 months of age was remarkably effective in inhibiting the expression and accumulation of CHI3L1, ACE2, and SPP in the older animals (Figure 5B). Although 12-month-old mice are the age equivalent of an approximately 40-year-old human, these studies demonstrate that the increase in circulating CHI3L1 that is seen even with this level of aging stimulates the expression and accumulation of ACE2 and SPP. They also suggest that these changes in CHI3L1, ACE2, and SPP contribute to the pathogenesis of the heightened COVID-19 responses in the elderly.

### Circulating CHI3L1 in COVID-19.

**Figure 6:**
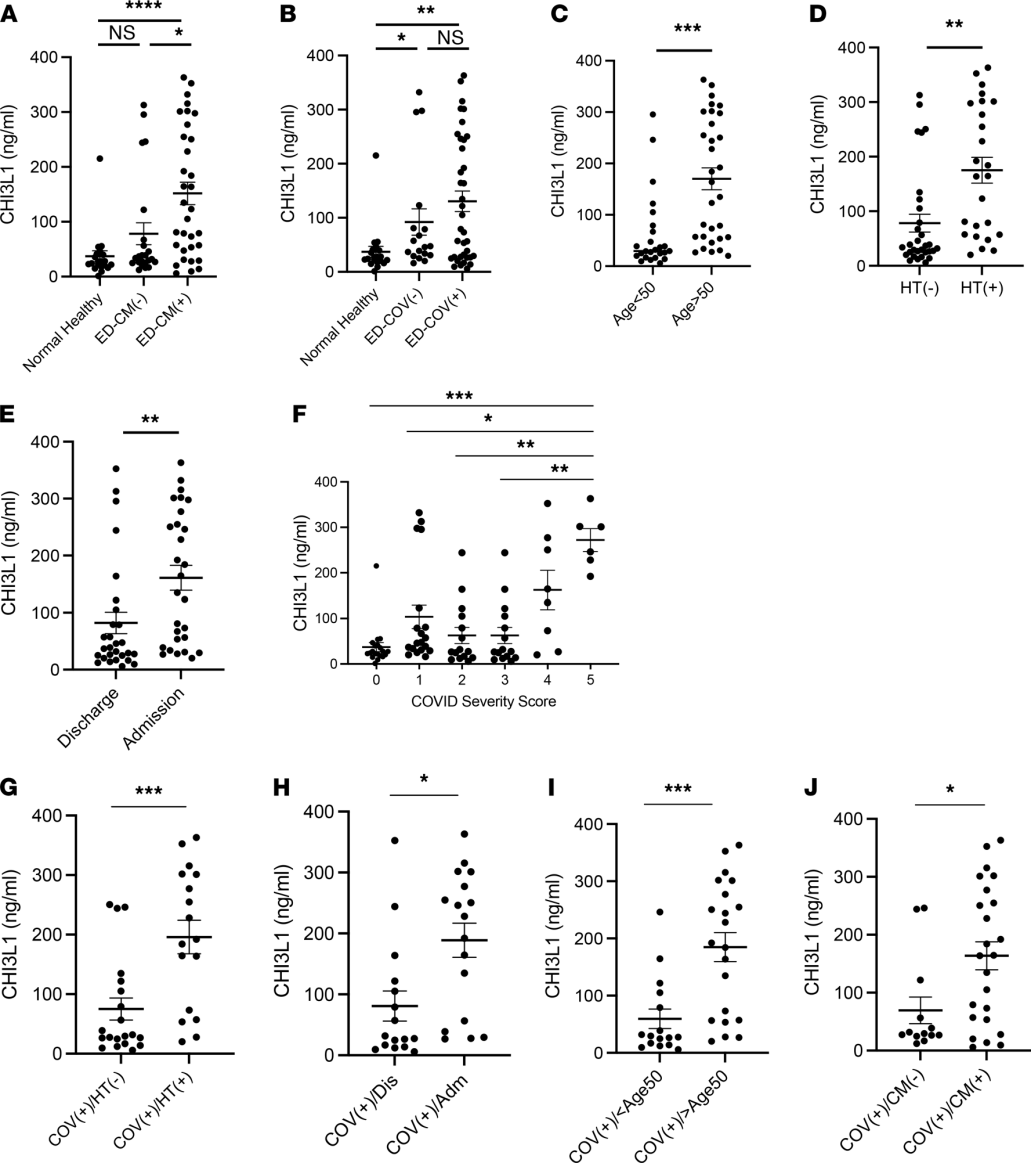
The levels of CHI3L1 in the circulation of patients with risk factors for COVID-19. The levels of CHI3L1 were assessed by ELISA using plasma from normals without comorbid diseases and patients presenting for emergency department evaluation. The values that were obtained were compared with the clinical features of the patients and the course of their diseases. Each value is from a different individual, and the mean ± SEM is illustrated. *P < 0.05, **P < 0.01, ***P < 0.001, ***P < 0.0001. **A**, **B**, and **F** used Kruskal-Walis with Dunn’s post hoc test for multiple comparisons; **C**–**E** and **G**–**J** used Mann-Whitney *U* test. Global *P* value of **F** was 0.0002. ER, emergency room; CM, comorbid disease; HT, hypertension; COV, COVID-19; Dis, discharge; Adm, admission; ICU, intensive care unit.

As noted above, SC2 infections are more common and more severe in the elderly and patients with comorbid diseases (1–3, 5, 16, 20, 21). Studies from our laboratory and others have also demonstrated that the levels of circulating CHI3L1 are also increased in the diseases that are COVID-19 risk factors (52–59). Thus, to further understand the relationships between CHI3L1 and the COVID-19 risk factors, we measured the levels of CHI3L1 in the serum of healthy individuals and patients presenting to the emergency department (ED) at Rhode Island Hospital (Providence, Rhode Island, USA) for medical evaluations that prompted a COVID-19 diagnostic evaluation. The demographics of these cohorts can be seen in Supplemental Table 1. In keeping with reports from our laboratory and others, the majority of the healthy individuals had levels of circulating CHI3L1 between 15 and 60 ng/mL (Figure 6A). Interestingly, the levels of circulating CHI3L1 in the individuals in the healthy cohort were not significantly different than the levels in patients presenting to the ED who did not have comorbid diseases (hypertension, diabetes, arthritis, neurologic disease, cancer, stroke, obesity, and/or chronic lung disease) (Figure 6A). The levels were, however, significantly lower than the levels in the circulation of patients presenting to the ED with existing comorbid diseases (Figure 6A) and patients who tested positive for COVID-19 (Figure 6B). When the COVID-19^–^ and COVID-19^+^ ED patients were evaluated together, the levels of circulating CHI3L1 were increased in the patients who were older than 50 years of age (Figure 6C) and/or had hypertension (Figure 6D). The levels of circulating CHI3L1 correlated with COVID-19 disease severity. This can be seen in Figure 6, E and F, which demonstrates that the levels were significantly increased in patients who were admitted to the hospital compared with those who were discharged to home (Figure 6E); it also demonstrates that the circulating levels of CHI3L1 were significantly increased in patients with higher COVID Severity Scores (CSS) (Figure 6F and Supplemental Table 2). When only COVID-19^+^ patients were evaluated, the levels of circulating CHI3L1 were increased in patients with hypertension versus those without hypertension; patients who were admitted versus those who were sent home from the ED; patients who were older than 50 years old versus younger individuals; and patients with comorbid diseases versus COVID-19^+^ patients without concurrent comorbid disorders (Figure 6, G–J). Because comorbidities occur with increased frequency as patients age, efforts were undertaken to disentangle the contributions of aging and comorbid diseases to the levels of circulating CHI3L1. This was done using 1-way univariate analysis of covariance (ANCOVA). These evaluations determined the statistical significance of comorbidity as an independent variable for the dependent variable CHI3L1. They also demonstrate that age is a covariate. Because the statistical differences in the levels of CHI3L1 in comparisons of individuals with and without comorbidity adjusted by age were still significantly maintained in this covariance analysis (*P* = 0.038), we consider comorbidity and age to be independent variables contributing to the levels of circulating CHI3L1.

Overall, these studies demonstrate that the levels of circulating CHI3L1 are increased in the elderly and patients with comorbid disease like hypertension. They also demonstrate that the levels of circulating CHI3L1 are increased in COVID-19^+^ patients who are elderly, have comorbid diseases, and manifest severe COVID-19. These observations support the concept that SC2 co-opts the CHI3L1 axis to stimulate ACE2 and SPP, which augment viral infection and foster SC2 disease manifestations. They also suggest that the effects of the COVID-19 risk factors are mediated, at least in part, by their ability to stimulate CHI3L1.

## Discussion

To determine if the CHI3L1 axis, ACE2, and SPP are related to one another, we characterized the effects of CHI3L1 on ACE2 and SPP in vivo and in vitro. In our initial studies, we compared the expression of ACE2 and SPP in lungs from CC10-driven CHI3L1-overexpressing Tg mice and WT controls. These studies demonstrate induction of ACE2, TMPRSS2, and CTSL in lungs from Tg^+^ mice. Ace2 was most prominent in airway epithelium. It was also seen in alveoli, where it colocalized with SP-C and CC10, and stained blood vessels, where it colocalized on endothelial and smooth muscle cells. TMPRSS2 and CTSL were also seen in airway epithelial cells, and TMPRSS2 was seen in alveolar macrophages. Endogenous CHI3L1 was also demonstrated to be significant because the levels of expression of ACE2 and SPP were decreased in comparisons of lungs from CHI3L1-null and WT mice. In the in vitro experiments, rCHI3L1 impressively stimulated the levels of mRNA encoding ACE2, TMPRSS2, and CTSL in human lung epithelial cell lines (Calu-3, A549) and primary HSAECs and lung fibroblasts. Importantly, these inductive responses were markedly decreased by treatment with moieties that alter CHI3L1-induced effector responses, such as monoclonal anti-CHI3L1 (FRG), kasugamycin, and inhibitors of CHI3L1 phosphorylation. These findings support the concept that SC2 co-opts the CHI3L1 healing and repair response to increase the number of cellular targets for viral entry. They also suggest that the host’s attempt to heal and repair via CHI3L1 is complicit in SC2 viral infection and COVID-19 morbidity and mortality.

In the time since the outbreak of the COVID-19 pandemic, unique features of its epidemiology have been appreciated. This includes the impressive increase in the prevalence and severity of the disease in the elderly, particularly those in congregate care settings (17, 18). The prevalence and severity of COVID-19 are also increased in patients with comorbid diseases including diabetes, hypertension, obesity and metabolic syndrome, cardiovascular disease, and chronic lung diseases like COPD, where they associate with enhanced susceptibility to SC2 infection, more severe disease, and a higher mortality (1–3, 5, 16, 20, 21). These unique features have interesting parallels in CHI3L1. Specifically, the levels of circulating CHI3L1 increase with aging. In fact, they have been reported to be the best predictor of all-cause mortality in octogenarians (64). In addition, studies from our laboratory and others have demonstrated that the levels of circulating CHI3L1 are also increased in the very diseases that are COVID-19 risk factors (52–59), where they correlate with disease severity (54, 55, 58). ACE2 expression and viral loads have been suggested to increase with aging, and it has been proposed that these changes can explain the impressive severity of COVID-19 in the elderly and patients with comorbid diseases (17, 18). Our study supports this contention and provides insights into the mechanisms that may underlie these associations by demonstrating that the levels of circulating CHI3L1 increase during murine aging, that these inductive events are abrogated by treatment with anti-CHI3L1, and that CHI3L1 stimulates ACE2 and SPP, which increases ACE2-S protein-cell interaction, S protein activation, and pseudovirus-S infection.

COVID-19–associated vasculitis and vasculopathy are now considered defining features of the systemic disease caused by SC2 (11, 65). The importance of vascular alterations can also be appreciated in recent studies comparing the lungs of patients infected with SC2 and influenza (14). Both manifest diffuse alveolar damage and perivascular infiltrating lymphocytes. There were, however, distinctive vascular manifestations in the COVID-19 tissues. They included (a) severe endothelial injury with intraendothelial SC2 and disrupted endothelial membranes; (b) widespread vascular thrombosis; and (c) elevated levels of intussusceptive angiogenesis. They also noted increased numbers of ACE2^+^ endothelial cells in infected patients versus controls (14). These and other studies have led to the belief that endothelial injury plays a key role in these responses and that the injury is caused by direct viral infection and perivascular inflammation (11, 14). Our studies support this concept by demonstrating, for the first time to our knowledge, that CHI3L1 is a powerful stimulator of endothelial cell and vascular smooth muscle cell ACE2 and SPP. They also raise the intriguing possibility that the CHI3L1 is a key stimulator of the pulmonary and systemic manifestations of COVID-19 — and that it does this, at least in part, by enhancing the susceptibility of endothelial cells and smooth muscle cells to infection with the virus.

Studies from our lab and others have demonstrated that CHI3L1 is a critical regulator of inflammation and innate immunity and a stimulator of type 2 immune responses, fibroproliferative repair, and angiogenesis (39, 42, 43, 66–70). These studies also demonstrated that CHI3L1 is dysregulated in a variety of diseases characterized by injury, inflammation, and/or tissue remodeling (36–38, 42, 44–51). In keeping with these findings, we focused our recent efforts on the development of CHI3L1-based interventions that could serve as therapeutics for CHI3L1-dependent diseases. These studies have defined an exciting therapeutic platform based on CHI3L1. Our initial studies focused on biologic approaches and the generation of a panel of monoclonal antibodies against CHI3L1 using full-length and peptide immunogens. These antibodies were assessed in a variety of murine models. The most impressive responses were obtained with a monoclonal antibody against a peptide that contained the sequence between aa 223 and 234 of human CHI3L1. This antibody is now called FRG. It was originally a mouse IgG2b κ. A humanized version has been generated on an IgG1 backbone. There are a number of reasons to believe that FRG can be an effective therapeutic in COVID-19. First, FRG is a potent inhibitor of the basal levels and the CHI3L1-stimulated expression and accumulation of ACE2 and SPP. It is also important to point out that studies from our lab and others have demonstrated that CHI3L1 drives inflammation in a type 2 direction and augments type 2 and decreases type 1 T cell differentiation (39, 71). This is problematic for COVID-19 because, in contrast to type 2 immunity, type 1 immune responses have potent antiviral effects (72). Thus, FRG treatment would abrogate CHI3L1 stimulation of type 2 responses and augment type 1 antiviral responses in a number of cells and tissues. FRG also inhibits fibroproliferative repair, which could ameliorate the pathologic fibrosis that occurs in patients with respiratory failure due to COVID-19 who are intubated for an extended interval (73). Based on these observations, one can see how FRG could be used as a prophylactic to diminish the chances of an individual getting infected after exposure to a SC2-infected person. One can also see how FRG, alone or in combination with antivirals such as remdisivir, could be therapeutically useful in patients with established SC2 infection.

CHI3L1 can have protective and pathologic effects. Examples of the former include studies from our laboratory and others that demonstrated that CHI3L1 inhibits hyperoxia-induced acute lung injury (41, 74, 75), is a powerful inhibitor of cellular apoptosis (39), and controls bacterial infection via the regulation of inflammasome activation (76). Our studies demonstrated that CHI3L1 is a potent stimulator of type 2 inflammation, M2 macrophage differentiation, and tissue remodeling and that CHI3L1 excess is a major contributor to these responses in allergic and fibrotic disorders (39, 67, 77, 78). The present studies highlight the importance of CHI3L1 as a stimulator of ACE2 and SPP and demonstrate that these inductive events enhance tissue inflammation and injury in COVID-19. When viewed in combination, these findings highlight the critical roles that the homeostatic regulation of CHI3L1 play in the biology of healing, repair, and disease. It is important to point out, however, that our studies and studies from others have demonstrated that CHI3L1 needs to be controlled but does not need to be eliminated to control its contributions in pathologic settings. Our studies demonstrate that CHI3L1 can be neutralized with appropriate antibodies or antagonists or totally knocked out using systemic genetic methodologies without generating tissue and/or organ toxicities or infertility (39). Thus, we believe interventions that control the expression of CHI3L1 can decrease the augmented ACE2 expression that is seen during aging and, by so doing, can diminish the consequences of SC2 infection without eliminating the protective effects of CHI3L1. Additional investigation will be required to determine if this hypothesis is correct.

CHI3L1 is known to promote type 2 immune responses such as those that are seen in allergic asthma, and IL-13 plays a key role in these responses (39). IL-13 and other Th2 cytokines are also known to inhibit the expression of ACE2 (79). However, it is important to point out that the roles of type 2 cytokines and the effects of asthma on SC2 infection are somewhat controversial. Epidemiologic studies have reported that patients with asthma are protected from COVID-19 (80–82). Surprisingly, the importance of IL-13 and Th2 inflammation in this purported protection have not been defined. In contrast, recent studies reported that IL-13 is actually a driver of COVID-19 severity (83) and that, in SC2-infected mice, IL-13 neutralization decreases mortality and disease severity without altering viral load (83). When viewed in combination, our studies and the literature demonstrate that the expression of ACE2 is highly regulated. We understand how one could interpret the inhibition of ACE2 by IL-13 and/or IL-4 and the stimulation of ACE2 by CHI3L1 as contrasting events. However, we believe that is not the proper interpretation. We think that, instead, these events represent different facets of an integrated regulatory system that can stimulate and inhibit ACE2. Specifically, we believe IL-13 and/or IL-4 inhibit ACE2 while simultaneously stimulating CHI3L1, which, in turn, feeds back to stimulate ACE2. It is tempting to also speculate that this feedback loop is at least partially receptor mediated because CHI3L1 and IL-13/IL-4 both bind to and can utilize IL-13Rα2 and CD44 to mediate their effector responses (70, 84). Because IL-13Rα2 is a signaling moiety for CHI3L1 (70) and a decoy receptor for IL-13 (85), it is tempting to speculate that the different effects of IL-13/IL-4 and CHI3L1 on ACE2 are the result of the different interactions of CHI3L1 and IL-13/IL-4 with IL-13Rα2 and CD44.

Kasugamycin was isolated from *Streptomyces kasugaensis* in 1965 (86). It was initially appreciated to inhibit the growth of fungi, later noted to have modest antibacterial properties, and most recently shown to inhibit influenza and other viral infections (87). This prompted us to undertake experiments designed to determine if kasugamycin altered the effects of CHI3L1. These studies demonstrated that kasugamycin is a powerful inhibitor of CHI3L1 induction of epithelial ACE2 and SPP. Other studies also demonstrated that kasugamycin inhibits type 2 immune responses and pathologic fibrosis (unpublished observation). The ability of kasugamycin to ameliorate CHI3L1 induction of ACE2 and SPP while exerting antiviral and antifibrotic effects and augmenting type 1 immune responses suggests that it is a useful agent that can be used as a prophylactic or therapeutic in COVID-19. This is an interesting prospect because kasugamycin has been used as an antibiotic in humans with minimal complications (88) and in agricultural settings where EPA evaluation has demonstrated an impressive lack of toxicity (89).

Our studies demonstrate that CHI3L1 has an activated state that is phosphorylated and that this activation is mediated by CDKs. They also demonstrate that aa 230 plays a critical role in CHI3L1 effector responses. In keeping with these findings flavopiridol, a broad spectrum CDK inhibitor, proved to be a powerful inhibitor of CHI3L1 stimulation of ACE2 and SPP in vitro and in vivo. This is likely due, in great extent, to the inhibition of CDK 1 and/or 2 because BMS 265246 had similar effects. It is also tempting to speculate that, because the monoclonal antibody FRG was generated against a site of phosphorylation, its impressive potency is the result of its ability to function as a blocking antibody and its ability to interfere with this critical phosphorylation site. These findings suggest that flavopiridol, BMS 265246, and possibly other CDK inhibitors could be useful therapeutics in COVID-19. This could be an exciting example of drug repurposing because flavopiridol, which is also called Alvocidib, has already received orphan drug designation from the FDA and European Medicines Agency for the treatment of chronic lymphocytic leukemia (90).

RNA viruses often trigger the retinoic acid–inducible gene–like (RIG-like) helicase (RLH) innate immune system. In this response, antiviral immunity is triggered by the dsRNA and 5′ triphosphate–bearing molecules that arise in the cytosol as viral replication intermediates. These moieties are recognized by widely expressed cytoplasmic sensors from the RIG-I–like receptor family including RIG-I and melanoma differentiation–associated protein 5 (mda-5) (30). The RIG-I receptor with its attached replication intermediate then interacts with mitochondrial MAVS (mitochondrial antiviral signaling molecule), the central integrator of this pathway, to triggers a type I IFN–based antiviral response (30). The RLH pathway plays a major role in viral control and clearance. Interestingly, studies from our laboratory have also demonstrated that the RLH pathway is also a powerful inhibitor of CHI3L1 expression and accumulation (91). Based on these observations, one would expect that SC2 infection and subsequent RLH activation would decrease the levels of circulating CHI3L1. This would decrease the expression of ACE2 and SPP, which would decrease viral infection and spread, along with subsequent tissue injury. In keeping with the understanding that viruses have evolved strategies that suppress or evade antiviral immunity (92), the levels of induction of type I IFNs are decreased in patients with COVID-19 (93, 94). We believe this decreased production of type I IFNs is a major contributor to the prevalence and severity of COVID-19 because it would increase the levels of circulating CHI3L1, increase the expression and accumulation of ACE2 and SPP, and augment viral infection and spread.

Early strains of SC2 from Wuhan, China, showed limited genetic diversity (35). However, genetic epidemiologic investigations conducted in late February 2020 have identified an emerging D614G mutation of the SC2 S protein in viral strains from Europe (35, 95). Patients infected with D614G-associated SC2 manifest enhanced viral loads. Pseudotyped virus with G614 mutations also manifest enhanced viral infectivity based on a S protein that is more likely to assume an “open” configuration and bind to ACE2 with enhanced avidity (35). One can envision how S protein mutations can lead to viruses that are increasingly problematic from a therapeutic perspective. However, one can also see how CHI3L1-based therapeutics that inhibit ACE2 and SPP can inhibit SC2 infection regardless of the S protein sequence.

Our studies demonstrate that CHI3L1 is a major stimulator of ACE2 and SPP that enhances SC2 S protein–receptor binding and activation — and augments SC2 infection and spread. Our studies also led us to appreciate that the levels of circulating CHI3L1 play a major role in defining the propensity for and severity of SC2 infection and contribute to the mechanisms by which aging and comorbid diseases contribute to the pathogenesis of COVID-19. To further understand the interactions between CHI3L1 and SC2, we measured the levels of circulating CHI3L1 in a cohort of healthy individuals and in a cohort of patients presenting to the ED with symptoms suggestive of COVID-19. These studies support our speculations in a number of ways. First, the levels of circulating CHI3L1 in patients in the ED cohort who were infected with SC2 and patients with comorbid diseases were significantly greater than in the healthy controls. In accord with what has been reported in the literature (64, 96), when the ED cohort patients were evaluated as a group, the levels of circulating CHI3L1 were increased in patients who were elderly, had hypertension, had comorbid diseases, or required hospitalization. Importantly, within the COVID-19^+^ ED patients, the levels of circulating CHI3L1 were statistically increased in the patients who were elderly, had hypertension or other comorbid diseases, and/or required hospitalization. These observations support the concept that SC2 co-opts the CHI3L1 axis to augment viral infection and foster its disease manifestations. They also raise the interesting possibility that quantification of the levels of circulating CHI3L1 can be useful in assessing the severity and need for hospital admission of patients presenting with COVID-19. Additional experimentation will be required to assess this speculation and further understand the relationships between CHI3L1 and COVID-19.

## Methods

### Genetically modified mice.

Lung-specific CHI3L1-overexpressing Tg mice in which CHI3L1 was targeted to the lung with the CC10 promoter (CHI3L1 Tg) have been generated and characterized by our laboratory as previously described (39, 97). These mice were between 6 and 12 weeks old when used in these studies. All animals were humanely anesthetized with ketamine/xylazine (100 mg/10 mg/kg/mouse) before any intervention.

### Western blot analysis.

Protein lysates from macrophages and whole mouse lungs were prepared with RIPA lysis buffer (Thermo Fisher Scientific) containing protease inhibitor cocktail (Thermo Fisher Scientific) as per the manufacturer’s instructions. In total, 20–30 μg of lysate protein was subjected to electrophoresis on a 4%–15% gradient mini-Protean TGX gel (Bio-Rad). It was then transferred to a membrane using a semidry method with a Trans-Blot Turbo Transfer System (Bio-Rad). The membranes were then blocked with Tris-buffered saline with Tween 20 (TBST) with 5% nonfat milk for 1 hour at room temperature. After blocking, the membranes were incubated with the primary antibodies overnight at 4°C in TBST and 5% BSA. The primary antibodies used in this study were α-ACE2 (Abcam, ERR4435[2], catalog ab108252), α-TMPRSS2 (Abcam, EPR3861, catalog ab92323), α-CTSL (R&D Systems, catalog AF1515), and β-actin (Santa Cruz Biotechnology, catalog sc47778). The membranes were then washed 3 times with TBST and incubated with secondary antibodies in TBST, 5% nonfat milk, for 1 hour at room temperature. After 3 additional TBST washes, Supersignal West Femto Maximum Sensitivity Substrate Solution (Thermo Fisher Scientific) was added to the membrane, and immunoreactive bands were detected by using a ChemiDoc (Bio-Rad) imaging system. The serine phosphorylation of CHI3L1 was detected by immunoblots of recombinant CHI3L1 with α-phosphoserine antibody (EMD Millipore, AB1603). For relative quantitation of the band intensity, densitometry data were generated from all of the Western blot images (Supplemental Figure 6).

### Cell lines and primary cells in culture.

Calu-3 (HTB-55) and A549 (CRM-CCL-185) lung epithelial cells were purchased from American Tissue Type Collection (ATCC) and maintained at 37°C in DMEM supplemented with high glucose, l-glutamine, minimal essential media (MEM) nonessential amino acids, penicillin/streptomycin, and 10% FBS until used. The primary HSAECs (ATCC, PCS-3010010) were maintained in airway epithelial cell basal media (ATCC, PCS-300-030) supplemented with bronchial epithelial cell growth kit (ATCC, PCS-300-040) components according to the protocol provided by ATCC. The primary normal human lung fibroblasts (NHLF; ATCC, PCS-201-013) were maintained in fibroblast basal media (ATCC, PCS-201-030) supplemented with fibroblast growth kit (ATCC, PCS-201-041) components according to the protocol provided by ATCC.

### RNA extraction and RT-qPCR.

Total cellular RNA was obtained using TRIzol reagent (Thermo Fisher Scientific) followed by RNA extraction using RNeasy Mini Kit (QIAGEN) according to the manufacturer’s instructions. mRNA was measured and used for RT-qPCR as described previously (39, 41). The primer sequences used in these studies are summarized in Supplemental Table 3. Ct values of the test genes were normalized to internal housekeeping genes such as β-actin, GAPDH, or RPL13a.

### IHC.

FFPE lung tissue blocks were serially sectioned at 5 μm thickness and mounted on glass slides. After deparaffinization and dehydration, heat-induced epitope retrieval was performed by boiling the samples in a steamer for 30 minutes in antigen unmasking solution (Abcam, antigen retrieval buffer, 100× citrate buffer, pH 6.0). To prevent nonspecific protein binding, all sections were blocked in a ready-to-use serum-free protein blocking solution (Dako/Agilent) for 10 minutes at room temperature. The sections were then incubated with primary antibodies — α-Ace2 (R&D Systems, AF3437), α-Tmprss2 (Abcam, ab92323), α-Ctsl (R&D Systems, AF1515), α-CC10 (Santa Cruz Biotechnology, sc-365992), α-SPC (Abcam, ab90716), α-CD31 (BD Pharmingen, 550274), α-transgelin (Abcam, ab14106) — overnight at 4°C. After 3 washings, fluorescence-labeled secondary antibodies were incubated for 1 hour at room temperature. The sections were then counterstained with DAPI and coverslips were added.

### Double-label IHC.

Double-label IHC was employed as previously described by our lab (39).

### Generation of monoclonal antibodies against CHI3L1 (FRG).

The murine monoclonal anti-CHI3L1 antibody (FRG) was generated using peptide antigen (aa 223–234 of human CHI3L1) as immunogen through Abmart Inc. This monoclonal antibody specifically detects both human and mouse CHI3L1 with high affinity (*K_D_* = 1.1 × 10^–9^). HEK293T cells were transfected with the FRG construct using Lipofectamine 3000 (Invitrogen, L3000015). Supernatant was collected for 7 days, and the antibody was purified using a protein A column (Thermo Fisher Scientific, 89960). Ligand binding affinity and sensitivity were assessed using ELISA techniques.

### SC2 pseudovirus infection.

Pseudotyped SC2, which has a lentiviral core expressing GFP but with the SC2 S protein (expressing D614 or G614 S protein) on its envelope, were obtained from COBRE Center for Stem Cells and Aging established at Brown University and Rhode Island Hospital. A plasmid expressing vesicular stomatitis virus (VSV-G) protein instead of the S protein was used to generate a pantropic control lentivirus. SC2 pseudovirus or VSV-G lentivirus was used to spin-infect Calu-3 cells in a 12-well plate (931*g* for 2 hours at 30°C in the presence of 8 μg/mL polybrene). Fluorescence microscopic images were taken 18 hours after infection. Flow cytometry analysis of GFP^+^ cells was carried out 48 hours after infection on a BD LSRII flow cytometer and with the FlowJo software.

### Assessment of the effects of CHI3L1 on S protein-ACE2 binding and S protein processing.

The effects of CHI3L1 on the binding of ACE2 and S protein were evaluated using Calu-3 cells and rS protein. In brief, Calu-3 cells were incubated with vehicle (PBS) or rCHI3L1 (500 ng/mL) for 24 hours; rS protein from SARS-CoV-2 (GenScript, Z03481-100) was added to the cells and further incubated for 2 hours. The cells were then harvested and subjected to Co-IP and immunoblot assays as described by our lab (70). The impact of CHI3L1 on the cellular processing of S protein was also assessed using Calu-3 cells in vitro. In these experiments, cells were incubated with vehicle (PBS) or rCHI3L1 (500 ng/mL) for 24 hours and harvested, and lysates were produced. The lysates were then incubated with rS protein for 2 hours, and the uncleaved S and cleaved S1 and S2 proteins were evaluated by Western immunoblot analysis using anti-SC2 S antibody (Proscience, 3525).

### Generation of WT and mutant forms of rCHI3L1.

WT CHI3L1-histidine–tagged pcDNA was obtained from MedImmune Inc. The putative serine or tyrosine phosphorylation sites of CHI3L1 located between aa 230 and 239 were mutated using methods of site-directed mutagenesis. The plasmids containing WT and 4 mutant forms of CHI3L1 — M230 (Ser-Arg), M235 (Ser-Arg), M237 (Thr-Phe), M239 (Tyr-Arg) — were transfected to HEK293 cells (ATCC, CRL-1573), and each recombinant protein was purified using HisPur Ni-NTA column (Invitrogen, 88226). The specificity of recombinant protein was further validated by SDS-PAGE and Western blot evaluations.

### Biobank and healthy donor samples.

Deidentified COVID-19^+^ and COVID-19^–^ human plasma samples were received from the Lifespan-Brown COVID-19 Biobank at Rhode Island Hospital, Brown University. Normal, healthy, COVID-19^−^ samples were commercially available from Lee BioSolutions (991–58-PS-1, Lee BioSolutions).

### Clinical variables and CSS.

Available deidentified clinical variables were collected from patients and from chart review during their time in the ED. The following categorized variables were collected, including chronic diseases, chronic lung disease (such as asthma, COPD or emphysema), chronic kidney disease, chronic neurologic disease (such as Parkinson’s, Alzheimer’s, multiple sclerosis), heart disease, high blood pressure, autoimmune diseases (such as lupus or rheumatoid arthritis), HIV/AIDS, active cancer (currently on treatment), previous stroke, or overweight or obesity (overweight for height > 99 percentile). Symptoms were also assessed, including breathing difficulty or shortness of breath, fever, cough, change in taste or smell, rash, gastrointestinal symptoms (such as abdominal pain, vomiting, diarrhea), neurologic symptoms (including stroke-like symptoms), sore throat, and chest pain. A CSS was assigned to each individual ED patient based on their COVID-19 infection and their clinical status. This included their comorbid diseases, symptoms, discharge or admission, oxygen treatment, or ICU transfer as outlined in Supplemental Table 2.

### Statistics.

Statistical evaluations were undertaken with SPSS or GraphPad Prism software. As appropriate, groups were compared with 2-tailed Student’s *t* test or with nonparametric Mann-Whitney *U* test. Values are expressed as mean ± SEM. One-way ANOVA or nonparametric Kruskal-Wallis tests were used for multiple group comparisons. The potential effects between subjects were tested by covariance analysis (1-way ANCOVA, SPSS, Version 28.0.0.0). Statistical significance was defined as a level of *P* < 0.05.

### Study approval.

As the human samples analyzed in this study were provided by LifespanBrown COVID-19 Biobank or purchased from Lee BioSolutions, the IRB study protocol “Pilot Study Evaluating Cytokine Profiles in COVID-19 Patient Samples” did not meet the definition of human subject research by either the Brown University or the Rhode Island Hospital IRB. All animal procedures and experiments were conducted according to protocols approved by the IACUC at Brown University.

## Author contributions

Conception and design were contributed by SK, JAE, CGL, BM, YZ, and BA. Generation of experimental resources and data collection were contributed by SK, BM, CHH, BA, CML, WSED, KH, OL, MJK, HJS, and EM. Analysis and interpretation were contributed by SK, BA, BM, CML, CHH, JTM, EM, CGL, and JAE. Drafting the manuscript for important intellectual content was contributed by JAE and CGL.

## Supplementary Material

Supplemental data

## Version 1 11/08/2021

Electronic publication
