# Supplementary material for: Chitinase 3-like-1 is a therapeutic target that mediates the effects of aging in COVID-19
Source: JCI Insight. 2021 Nov 8;6(21):e148749. doi: 10.1172/jci.insight.148749 (PMC8663553; doi:10.1172/jci.insight.148749)
Supplement: Supplemental data [file jciinsight-6-148749-s051.pdf]

Supplementary Tables

Table S1. Demographic features of the healthy controls and a cohort of patients used in this study

	Healthy Control	ED Patients
Sex		
Male	10	32
Female	10	25
Age (mean±SD)*	48.9±15.9	51.2±20.25
Ethnicity		
White	15	18
Black	0	7
Hispanic/Latino	3	21
Asian or Pacific Origin	2	3
Other or unknown		7
COVID-19		
Negative	20	19
Positive	0	37

*, no significant difference between healthy control vs ED patients (t-test, p=0.65)

Table S2. COVID Severity Score

COVID Security Score		Sample Size
0	COVID (-) Healthy Control	20
1	COVID (-) ED Visit (Chronic Disease or symptoms)	19
2	COVID (+) ED Visit and Discharged	16
3	COVID (+) ED Visit and Admitted, No Oxygen	7
4	COVID (+) ED Visit and Admitted, Oxygen	8
5	COVID (+) ED Visit and Admitted to ICU	6

COVID, COVID-19; ED, Emergency Department; ICU, Intensive Care Unit

Table S3. Sequences of RT-PCR primers used in this study

Species	Gene	Sequence (5'to 3')
Mouse	Ace2-S	TCCAGACTCCGATCATCAAGC
	Ace2-AS	GCTCATGGTGTTCAGAATTGTGT
	Tmprss2-S	CAGTCTGAGCACATCTGTCCT
	Tmprss2-AS	CTCGGAGCATACTGAGGCA
	Ctsl-S	ATCAAACCTTTAGTGCAGAGTGG
	Ctsl-AS	CTGTATTCCCCGTTGTGTAGC
	β-Actin-S	GGCTGTATTCCCCTCCATCG
	β-Actin-AS	CCAGTTGGTAACAATGCCATGT
	RPL13a-S	AGGGGCAGGTTCTGGTATTG
	RPL13a-AS	TGTTGATGCCTTCACAGCGT
Human	ACE2-S	CGAAGCCGAAGACCTGTTCTA
	ACE2-AS	GGGCAAGTGTGGACTGTTCC
	TMPRSS2-S	GTCCCCACTGTCTACGAGGT
	TMPRSS2-AS	CAGACGACGGGGTTGGAAG
	CTSL-S	CTTTTGCCTGGGAATTGCCTC
	CTSL-AS	CATCGCCTTCCACTTGGTC
	β-ACTIN-S	GCCCTGAGGCACTCTTCCA
	β-ACTIN-AS	CGGATGTCCACGTCACACTTC
	GAPDH-S	GACAGTCAGCCGCATCTTCT
	GAPDH-AS	TTAAAAGCAGCCCTGGTGAC

Supplemental Figures

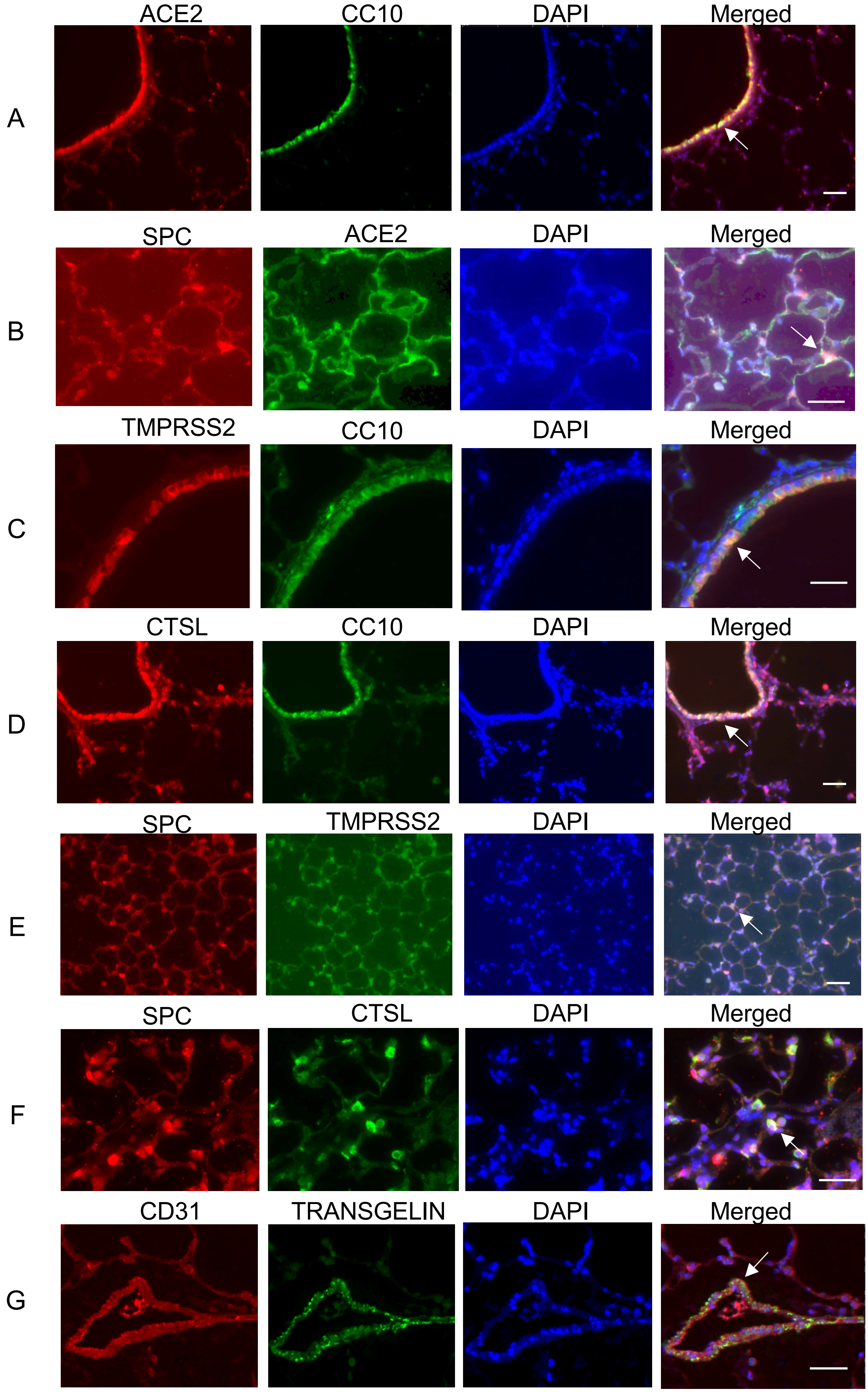

Figure S1. CHI3L1 induces the expression of pulmonary ACE2 and SPP. 8 weeks old *CHI3L1* Tg (+) mice were sacrificed after 2 weeks of transgene induction with Doxycycline. The cell specific expression of ACE2 and SPP were detected using double fluorescent immunohistochemistry. (A) Co-localization of ACE2 with airway epithelial marker of CC10 in the lungs of *CHI3L1* Tg mice. (B) Co-localization of ACE2 with airway type 2 alveolar epithelial marker of Pro-SPC in the lungs of *CHI3L1* Tg mice. (C-D) TMPRSS2 and CTSL with CC10 in the lungs of *CHI3L1* Tg mice. (E-F) Co-localization of TMPRSS2 and CTSL with pro-SPC in the lungs of CHI3L1 Tg mice. (G) Co-localization of ACE2 with vascular smooth muscle cell marker TRANSGELIN in the lungs of *CHI3L1* Tg mice. ACE2, murine angiotensin converting enzyme 2; TMPRSS2, transmembrane serine protease 2; CTSL, Cathepsin L. Scale bars=100μm.

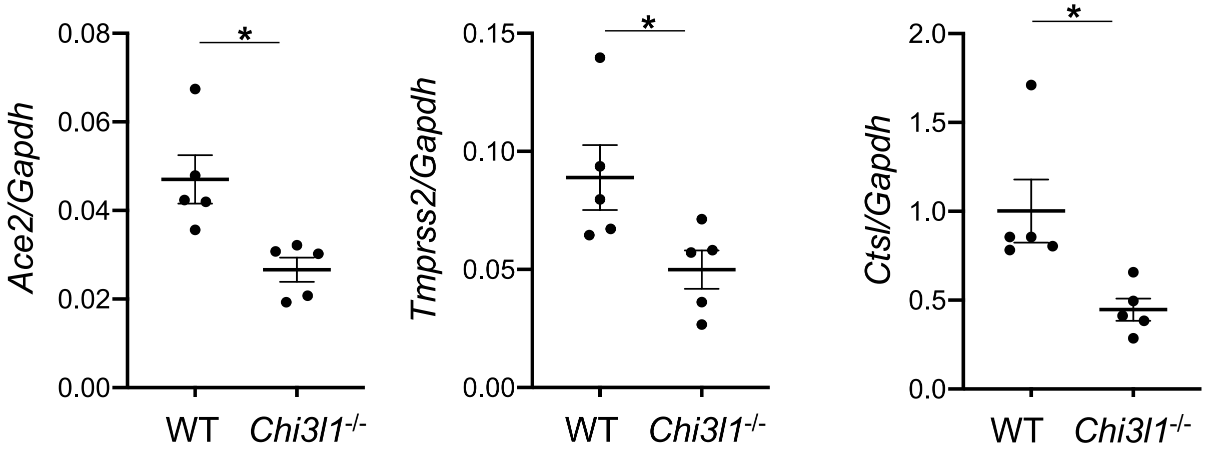

Figure S2. Role of CHI3L1 in endogenous expression of pulmonary ACE2 and SPP. 8 weeks old WT and *Chi3l1* null mutant (*Chi3l1*$^{-/-}$) mice were sacrificed and endogenous mRNA expression of Ace2, Tmprss2 and Ctsl were evaluated using real-time PCR. *p<0.05 (student *t*-test).

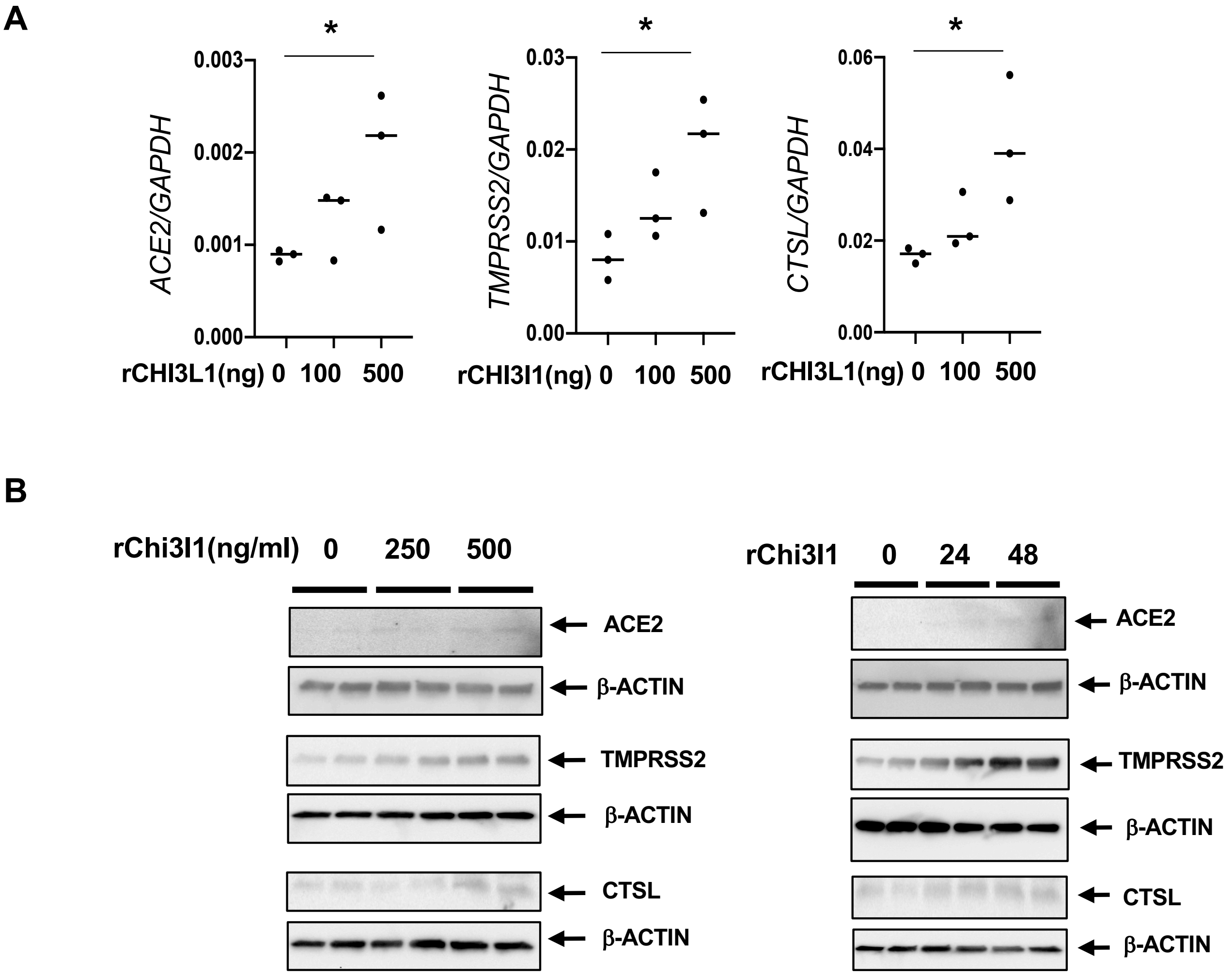

Figure S3. Chi3l1 stimulates the expression of ACE2, TMPRSS2 and CTSL in primary human airway epithelial cells. Human primary small airway epithelial cells (SAECS) were subjected to real time RT-PCR (A) and Western blot evaluations (B) after stimulation of the cells with recombinant human CHI3L1 in indicated dose and time points. Mean values are indicated. *p<0.05 (student *t*-test).

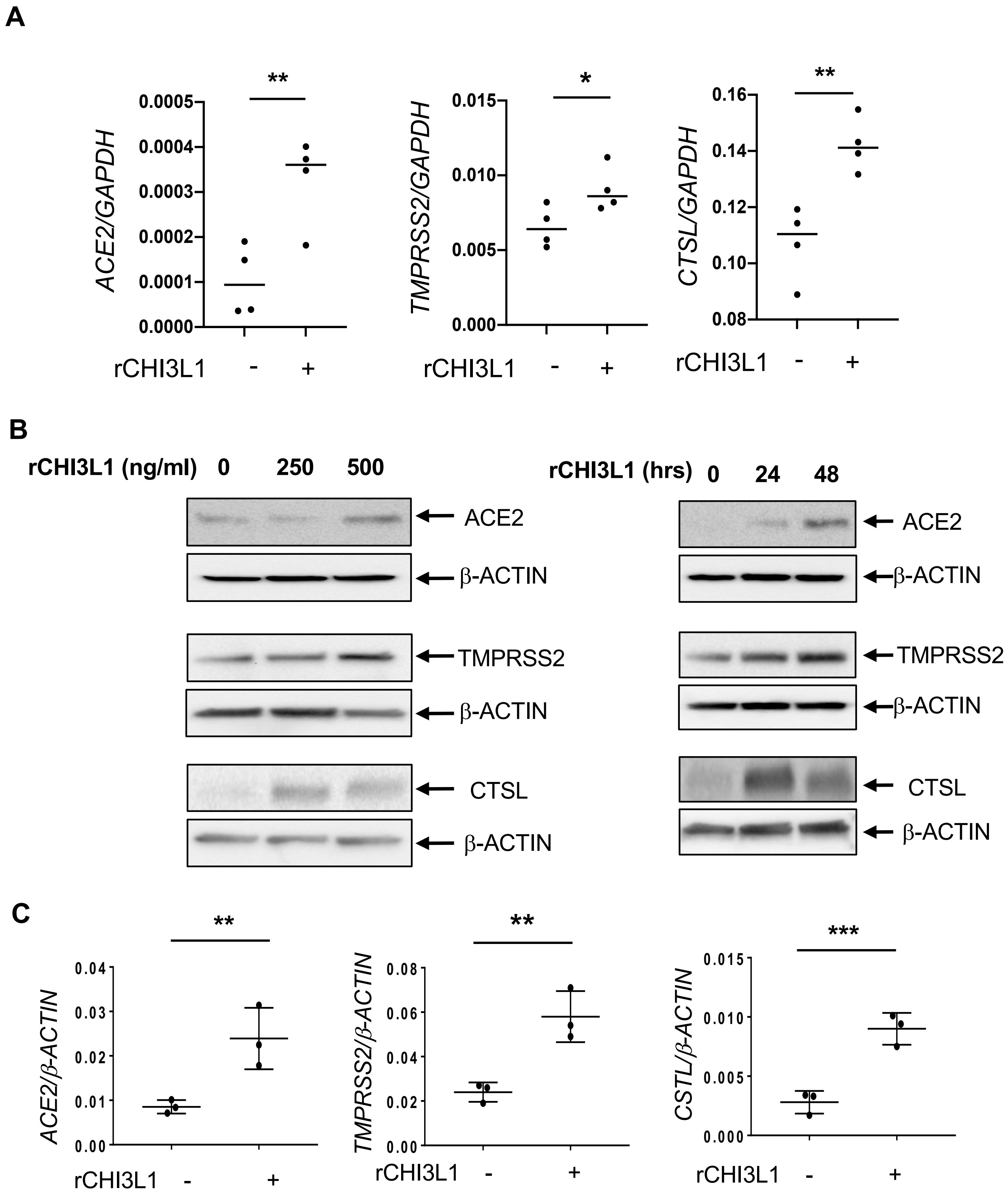

Figure S4. CHI3L1 stimulates the expression of ACE2, TMPRSS2 and CTSL in human airway epithelial cells and lung fibroblasts. (A-B) A549 lung epithelial cells were subjected to real time RT-PCR (A) and Western blot evaluations (B) after stimulation of the cells with indicated dose and time of rCHI3L1. (C) After stimulation of normal human lung fibroblasts (NHLF) with rCHI3L1 (250ng/ml) for 48 hours, the levels of mRNA expression of ACE2, TMPRSS2 and CTSL genes were evaluated by real time RT-PCR. Mean (panel A) and mean±SEM (panel C) are indicated. *p<0.05, **p<0.01, ***p<0.001 (student *t*-test)

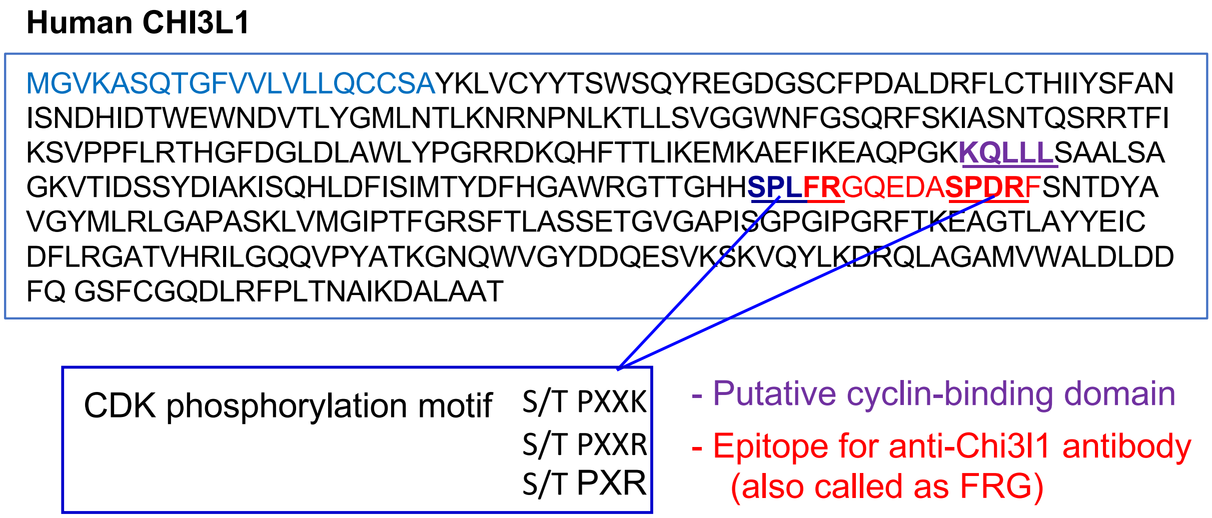

Figure S5. Prediction of CHI3L1 phosphorylation sites, cyclin binding domain, putative CDK activation site and illustration of the epitope used for anti-CHI3L1 antibody (FRG) generation.

A (Figure 1B)

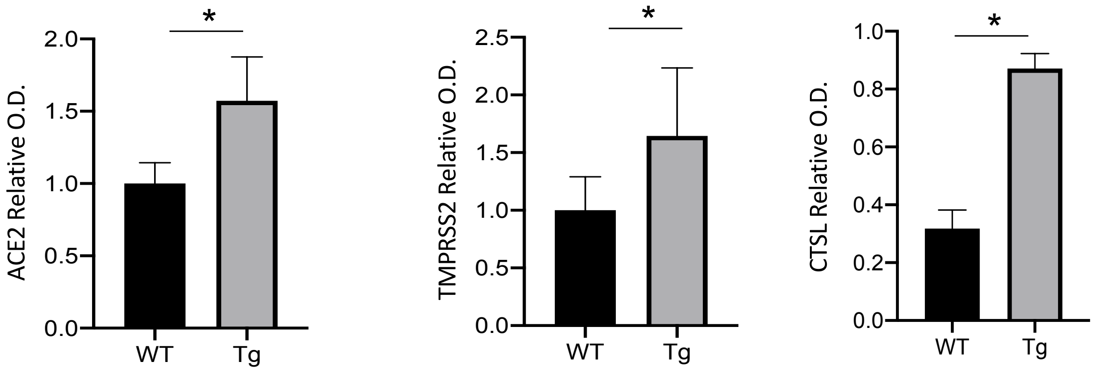

B (Figure 2B)

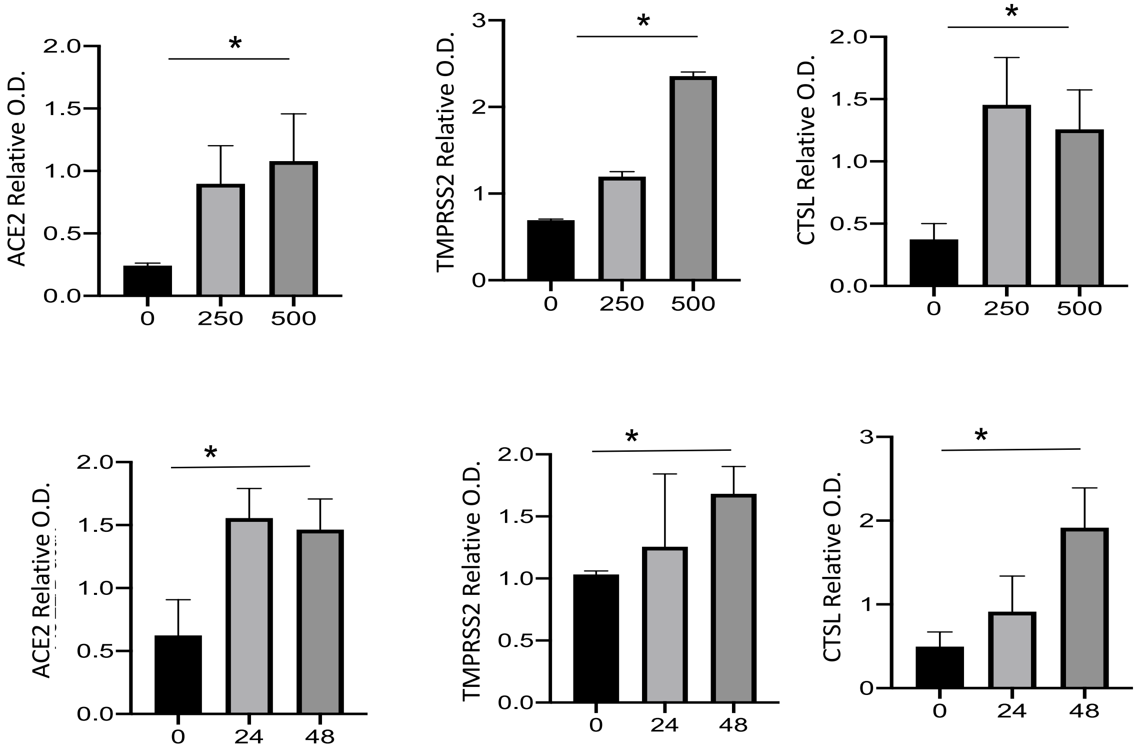

C (Figure 2C)

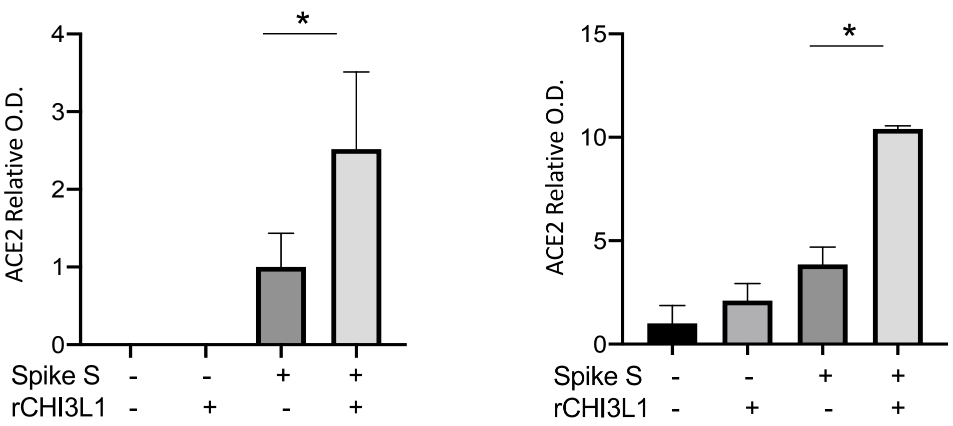

D (Figure 2D

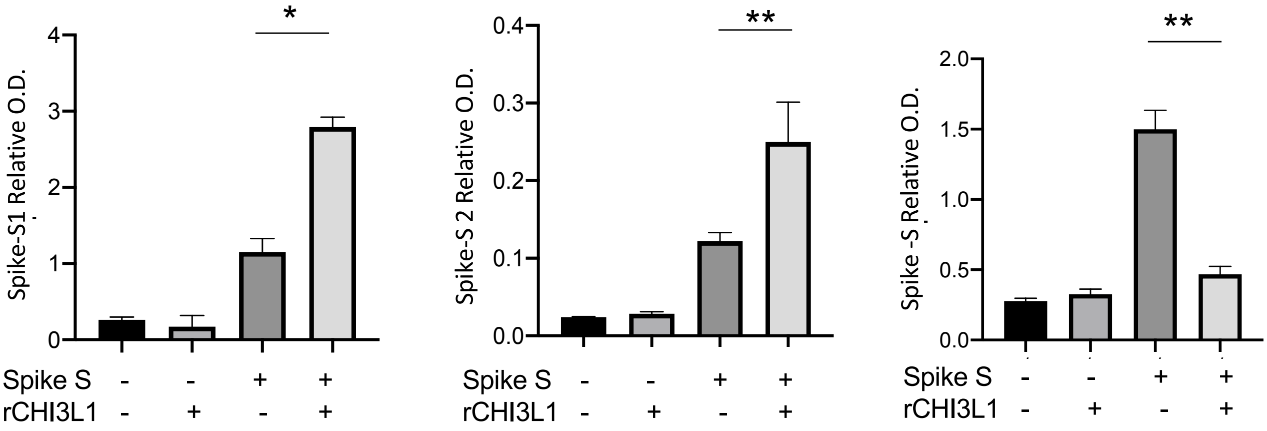

E (Figure 3B)

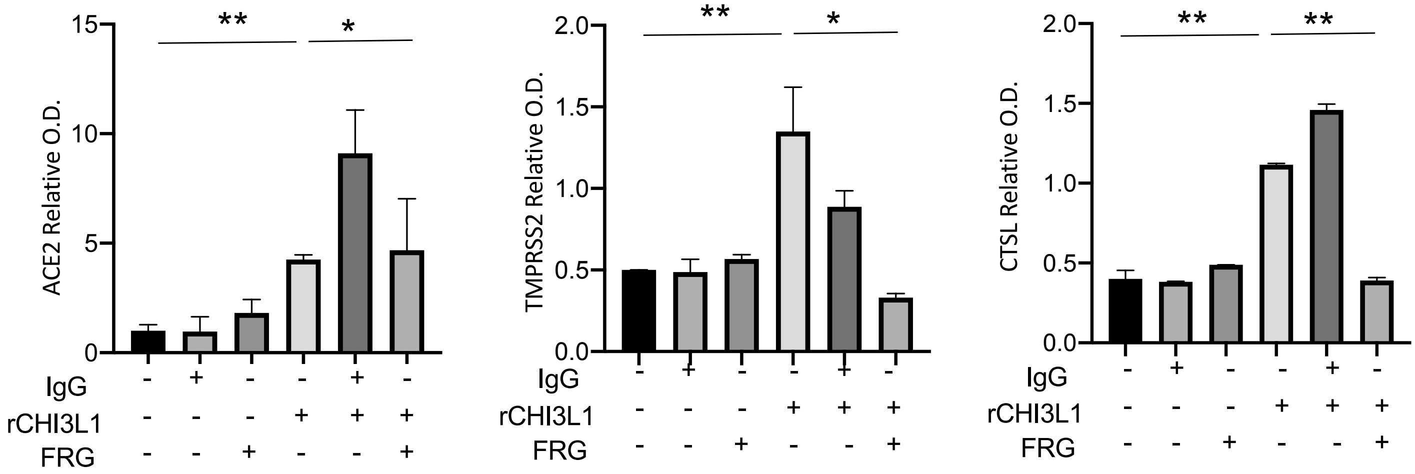

F (Figure 4A)

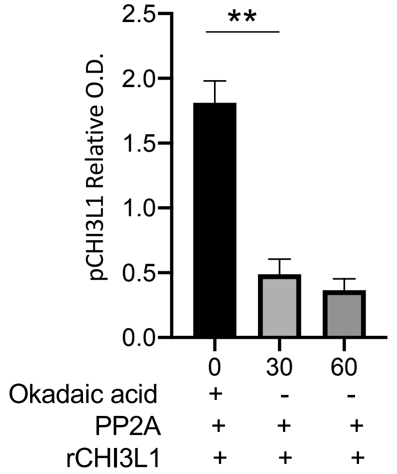

Figure S6. Semi-quantitation of the individual band intensity of the immunoblots included in main figures. Optical density was assessed by (Bio-Rad Image lab (v-5.2.1)) and relative ratio to control (or a reference lane) has been plotted and statistical significance was evaluated compared to controls. *P<0.05, **P<0.01 (*t*-test or ANOVA with multiple comparisons).
